# Risk Factors, Incidence, and Outcome of Stroke: A Retrospective Cross-Sectional Hospital-Based Study Comparing Young Adults and Elderly

**DOI:** 10.7759/cureus.40614

**Published:** 2023-06-19

**Authors:** Ameena Moosa, Dana Osama, Firas Alnidawi, Salman Algillidary, Ali Hussein, Priya Das

**Affiliations:** 1 Internal Medicine, King Hamad University Hospital, Muharraq, BHR; 2 Neurology, King Hamad University Hospital, Muharraq, BHR; 3 Scientific Research and Development, King Hamad University Hospital, Muharraq, BHR

**Keywords:** ischemic stroke, etiology, risk factors, hemorrhagic stroke, stroke in young adults

## Abstract

A noticeable increase of up to 40% in the incidence of stroke among young population over the past decade has been noted. This study aimed to investigate the incidence, risk factors, and outcomes of stroke and its subtypes in young adults compared to older population.

A retrospective study of patients which included patients with confirmed diagnosis of stroke based on the International Classification of Diseases 10th Revision (ICD-10) classification between the years 2018 and 2020 was conducted. The results indicated that patients less than 45 years of age had a higher incidence of hemorrhagic stroke as compared to the other age groups (p=0.011). Hypertension leading to hemorrhagic stroke was higher in patients less than 45 years of age as compared to other groups (18 years {19.4%} versus 33 years {7.5%}, p=0.001). Hypertension was noted to be the leading risk factor for stroke among the younger population.

## Introduction

Stroke has been traditionally considered a disease predominantly affecting the older population. However, there are some alarming trends that implied a noted increase in the incidence of stroke in younger people. Worldwide, more than two million young adults have had an ischemic stroke every year. In terms of defining young adults in the context of stroke, literature had variable lower and upper age limits. Some studies referred to this population as being between 18 and 50 years of age while others define them as between 18 and 45 years of age [1]. Over the past decade, there was an evident increase of up to 40% in the incidence of subarachnoid as well as intracerebral hemorrhagic stroke among younger population [1]. Of all first ever reported strokes an estimated 10-15% occur in younger adults [2,3].

Disparities in epidemiology of ischemic stroke among young adults have been noted based on geography and ethnicity with higher incidences noted in developing countries as compared to industrialized. Regrettably, in developing countries, young-onset strokes account for 30% of all strokes [4]. Whereas in Western countries, the incidence of strokes in a younger population range from 5% to 10% in subjects aged <45 years [4,5]. Reports indicate that the risk of ischemic stroke increase with age, however, the increase in incidences of these events among young adults poses a global burden [6]. Lack of data from many Asian and African countries contributes to the knowledge gap on global incidences of stroke among young adults.

Vascular risk factors, infections, and tobacco abuse were some of the commonly reported risk factors for stroke among the young population. Multiple potential etiologies of stroke in young adults have been described in the literature which include cervical artery dissections, hypercoagulable syndromes, patent foramen ovale (PFO), and rheumatic heart diseases, to mention a few. These risk factors are mostly seen to be commonly present in both older and younger populations with stroke. Although it is noted that 40% of acute ischemic strokes have no identifiable cause and are classified as cryptogenic ischemic strokes [6-8].

In addition to the above-mentioned risk factors, inherited and acquired thrombophilia can attribute to cryptogenic strokes, particularly in young adults. A retrospective cohort study of 196 patients aged 18-65 years concluded that two-of-five young patients with ischemic stroke who underwent thrombophilia screening had at least one positive test [9].

Furthermore, one of the most identifiable risk factors in young patient is patent foramen ovale (PFO), which was found to be almost isolated in young ages, these can result from an embolus in the venous system originating in the right atrium then transferred to the left atrium and into the systemic circulation through a PFO, a phenomenon known as paradoxical embolism [9,10].

Another frequently addressed risk factor is cervicocerebral arterial dissection with estimates suggesting that they account for 10-25% of all ischemic strokes in younger age groups [11]. Finally, regional studies highlight that the Bahraini stroke population was found to be 10 years younger than Western comparators with higher prevalence of traditional risk factors [12].

Primary and secondary prevention of stroke based on geographic/ethnic disparities leading to exposure to several vascular risk factors among young adults would require region-specific data on epidemiology and etiology of the event. Hence, the primary aim of this study was to address the incidence and risk factors of stroke in young patients and compare them to older population in the Kingdom of Bahrain.

## Materials and methods

Patients

The protocols of this study were in accordance with the guidelines of the Ethics Committee at King Hamad University Hospital, Bahrain. Through a hospital-based electronic medical record (EMR) system, all patients were identified for whom a code stroke in the emergency department was activated between 2018 and 2020. Only patients with a proven and radiologically identified stroke (ischemic stroke, intracerebral hemorrhage, or unspecified stroke) were included in this study. Patients who did not fulfill the criteria, in which symptoms were attributed to underlying metabolic disorders or brain tumors or stroke mimics like migraine and seizures, were directly excluded from the study. All available patient's data through EMR system was analyzed, including neuroimaging and outcomes upon discharge from hospitalization and subsequent clinic follow-up visits.

Case definition

Patients hospitalized with ischemic stroke, intracerebral hemorrhage, or other unspecified stroke were included in the study. Young adults were defined as those between the ages of 18 and 45 years at the time of stroke diagnosis, while the elderly age group included those aged ≥46 years.

Data analysis

Included patients in this review were initially divided into two main age subgroups (18-45 years and above 46 years). Data were entered in Excel and analyzed using SPSS version 25.0 (Armonk, NY: IBM Corp.). The data were categorized according to year, gender, ethnicity (Bahraini, Arab, Caucasian, Indian, Far Eastern, or African), subtype of stroke diagnosis (ischemic stroke, intracerebral hemorrhage, or unspecified stroke), and main contributing risk factors (diabetes, hypertension, dyslipidemia, underlying coronary artery disease or atrial fibrillation, previously diagnosed stroke or transient ischemic attack, peripheral arterial disease, and smoking). Descriptive statistics were used to compute the frequencies and percentages. Chi-square test was used to compare significant differences between two groups with categorical data. Relative risk was computed. All the statistical test was two-tailed, and a p-value of <0.05 was considered significant.

## Results

A total of 513 patients were reported with stroke during the study period, of which 93 (18.1%) were of age <45 years and 438 (82.4%) were of age >45 years. Patients less than 45 years of age had a higher incidence of hemorrhagic stroke as compared to the other age groups (21.5% versus 11.6%, p=0.011). The most incident risk factor in patients less than 45 years of age was hypertension (HTN) (43.0%), followed by diabetes mellitus (DM) (24.7%) and dyslipidemia (DL) (16.1%); a similar pattern was seen in patients greater than 45 years of age (HTN: 74.0%, DM: 63.0%, DL: 32.2%) (Table 1).

**Table 1:** Patient demographics (n=531).

Variables		Age <45 years	Age >45 years	p-Value
		(n=93)	(n=438)	
Diabetes mellitus (DM)	Yes	23 (24.7%)	267 (63.0%)	0
	No	70 (75.3%)	162 (37.0%)	
Hypertension (HTN)	Yes	40 (43.0%)	324 (74.0%)	0
	No	53 (57.0%)	114 (26.0%)	
Dyslipidemia (DL)	Yes	15 (16.1%)	141 (32.2%)	0.002
	No	78 (83.9%)	297 (67.8%)	
Coronary artery disease/atrial fibrillation (CAD/Afib)	Yes	6 (6.5%)	122 (27.9%)	0
	No	87 (93.5%)	316 (72.1%)	
Smoking	Yes	3 (3.2%)	22 (5.0%)	0.596
	No	90 (96.8%)	416 (95.0%)	
Peripheral artery disease (PAD)	Yes	0 (0.0%)	5 (1.1%)	0.593
	No	93 (100.0%)	433 (98.9%)	
Previous stroke/transient ischemic attack (TIA)	Yes	3 (3.2%)	114 (26.0%)	0
	No	90 (96.8%)	324 (74.0%)	
Independent and special care	Independent	91 (97.8%)	374 (85.8%)	0.015
	Special care at home	2 (2.2%)	58 (13.3%)	
	Nursing home	0 (0.0%)	3 (0.7%)	
Modified Rankin Scale (mRS)		0.09±0.66	0.90±1.59	0
Type of stroke	Ischemic	73 (78.5%)	381 (86.94%)	0.011
	Hemorrhagic	20 (21.5%)	51 (11.6%)	
	Both ischemic and hemorrhagic	0 (0.0%)	6 (1.3%)	
Outcome	Discharged home	54 (68.4%)	295 (68.3%)	0.24
	Transferred to other hospitals	23 (29.1%)	106 (24.5%)	
	Death	2 (2.5%)	31 (7.2%)	

**Figure 1:**
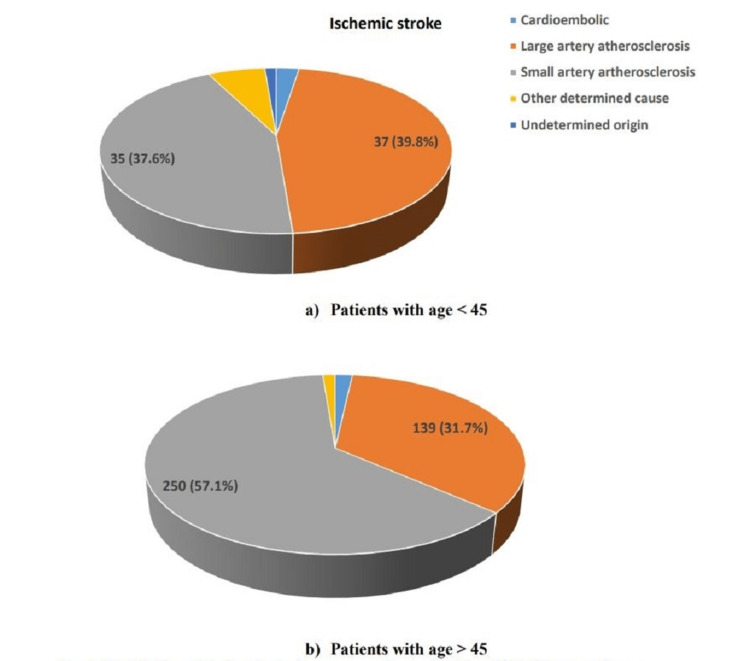
Distribution of ischemic stroke among patients (a) <45 and (b) >45 years of age.

**Figure 2:**
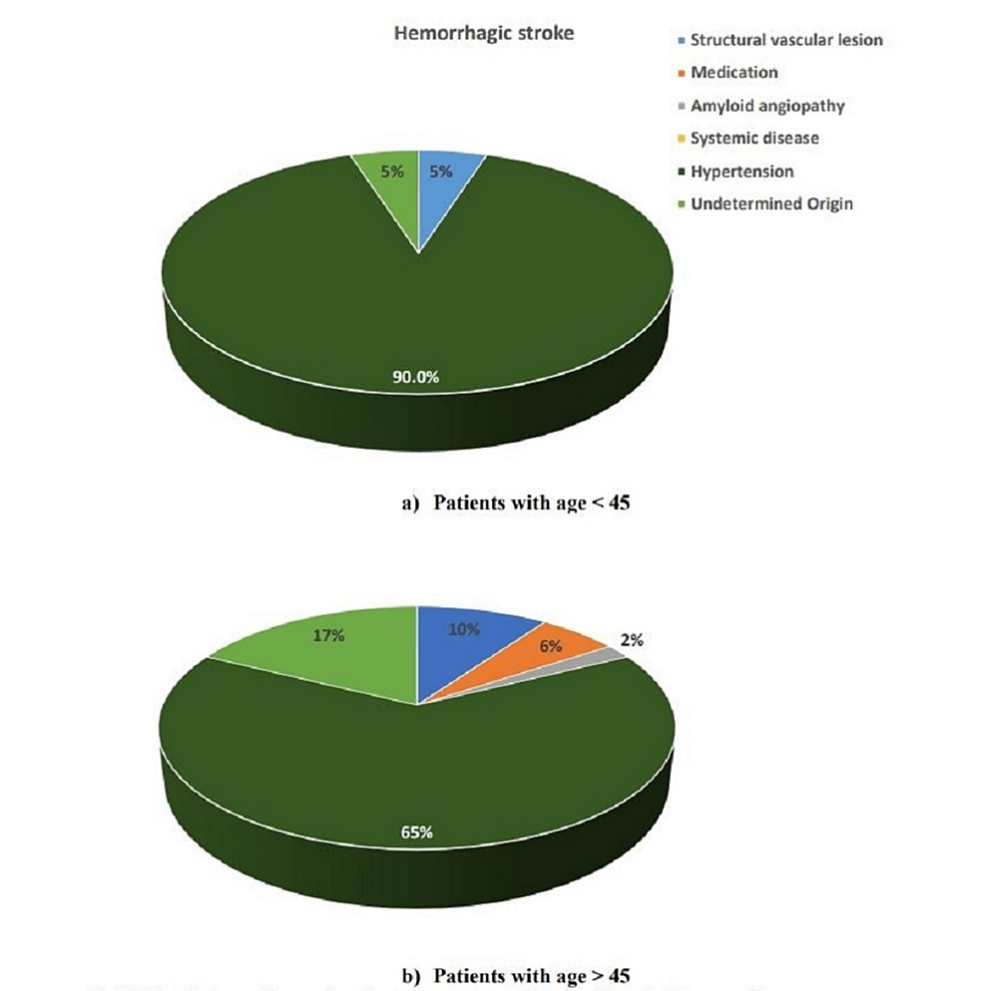
Distribution of hemorrhagic stroke among patients (a) <45 and (b) >45 years of age.

Thirty-seven (39.8%) patients less than 45 years of age had large artery atherosclerosis as compared to 139 (31.7%) in the group of patients above 45 years of age (Figure 1). Thirty-five (37.6%) patients less than 45 years of age had small artery atherosclerosis as compared to 250 (57.1%) in the other group. Hypertension leading to hemorrhagic stroke was higher in patients less than 45 years of age as compared to other groups (18 {90.0%} versus 33 {64.7%}, p=0.001) (Figure 2).

Outcome

Two mortality cases were reported in the age group of <45 years and both the patients (male: 38 years and female: 44 years) had ischemic stroke (large artery atherosclerosis). Male patient (38 years) had DM, HTN, and DL as comorbidities. The relative risk (RR) of mortality due to stroke is 3.2 times higher in patients >45 years of age as compared to younger group (RR: 3.29, 95% CI: 9.69-217.7, p=0.09), however, the finding was not significant.

## Discussion

Over a period of two years in a tertiary center stroke unit, 17% (n=93) of stroke patients admitted were under the age of 45 years, comparable to estimates of 10-15% in developing countries [13]. In patients under the age of 45 years, the incidence of stroke is usually considered to be relatively less than older patients. However, stroke incidence in young populations is rising due to the increasing trend of risk factors, such as hypertension, diabetes, and dyslipidemia which contribute to conventional vascular risk factors among this population. This trend is prevalent in developing countries due to their lifestyles, such as reduced exercise and abuse of polysubstance [7]. Increasing trends of conventional vascular risk factors are evident in this study as well. The most incident risk factor in patients less than 45 years of age was hypertension (43.0%), followed by diabetes mellitus (24.7%) and dyslipidemia (16.1%); a similar pattern was seen in patients greater than 45 years of age (HTN: 74.0%, DM: 63.0%, DL: 32.2%) [14]. Some of the rare causes such as arterial dissection, arteriopathy, PFO, genetic causes, and hypercoagulable state must be considered if stroke develops in this population without any conventional risk factors.

This retrospective study also showed that young patients had higher incidence of hemorrhagic stroke compared to older population. Higher incidence of hemorrhagic stroke can be explained by the increased prevalence of hypertension in young adults which if uncontrolled can lead to higher incidence of hemorrhagic strokes. It can also be explained by a decrease in prevalence of dyslipidemia when compared to older populations, as dyslipidemia leads to atherosclerotic disease which is a "ticking time bomb" to the development of ischemic strokes [7].

Looking into the findings of this study, the focus of the healthcare system should be targeted towards primary prevention of stroke, by targeting the most widely prevalent risk factors. In addition to recognizing the heterogeneity of the younger stroke population and developing a focused preventative strategy that will also address other less common causes [15].

Limitations and implications

The cohort size of this study is one of the limiting factors for this study. One way to address this is to continue monitoring data and to increase awareness across multiple centers as a way to increase power and external validity of future studies. Another limitation was the unavailability of proper documentation in regard to the smoking status of the patients. Smoking is one of the factors that could increase cardiovascular diseases, thus it must be considered that smoking might be a confounding variable in this study. Another limitation is the lack of accurate demographic characteristics like gender and ethnicity which could help us identify key patterns and to develop strategic preventative strategies to address the targeted population.

One of the strengths of this study is providing new data regarding stroke in young adults in the region. This can be the stepping stone to collecting more comprehensive and valuable data regarding stroke in young adults in our region. This will help increase awareness regarding the epidemiology of stroke by discovering trends in causality with non-communicable diseases [12,16,17].

## Conclusions

An increasing trend in the incidence of stroke is seen among young adults, the majority of the cases which is attributable to lifestyle risk factors may vary across regions around the globe. The outcomes of stroke can be disabling, hence stroke among young raises an alarming situation that has a significant impact on the economies of developing countries. Further large-scale studies need to be conducted to establish improved management strategies that could be region specific if required.

## References

[1] Ekker MS, Boot EM, Singhal AB, Tan KS, Debette S, Tuladhar AM, de Leeuw FE (2018). Epidemiology, aetiology, and management of ischaemic stroke in young adults. Lancet Neurol.

[2] Mackey J (2014). Evaluation and management of stroke in young adults. Continuum (Minneap Minn).

[3] Fan H, Hao X, Yang S (2018). Study on the incidence and risk factor of silent cerebrovascular disease in young adults with first-ever stroke. Medicine (Baltimore).

[4] Hathidara MY, Saini V, Malik AM (2019). Stroke in the young: a global update. Curr Neurol Neurosci Rep.

[5] (2023). Textbook of Stroke Medicine. Textbook of Stroke Medicine.

[6] von Sarnowski B, Putaala J, Grittner U (2013). Lifestyle risk factors for ischemic stroke and transient ischemic attack in young adults in the Stroke in Young Fabry Patients study. Stroke.

[7] Maaijwee NA, Rutten-Jacobs LC, Schaapsmeerders P, van Dijk EJ, de Leeuw FE (2014). Ischaemic stroke in young adults: risk factors and long-term consequences. Nat Rev Neurol.

[8] Omran SS, Lerario MP, Gialdini G (2019). Clinical impact of thrombophilia screening in young adults with ischemic stroke. J Stroke Cerebrovasc Dis.

[9] Steiner MM, Di Tullio MR, Rundek T (1998). Patent foramen ovale size and embolic brain imaging findings among patients with ischemic stroke. Stroke.

[10] Furlan AJ, Reisman M, Massaro J (2012). Closure or medical therapy for cryptogenic stroke with patent foramen ovale. N Engl J Med.

[11] Stack CA, Cole JW (2018). Ischemic stroke in young adults. Curr Opin Cardiol.

[12] Al Banna M, Baldawi H, Kadhim A, Humaidan H, Whitford DL (2015). Stroke in Bahrain: rising incidence, multiple risk factors, and suboptimal care. Int J Stroke.

[13] Smajlović D (2022). Strokes in young adults: epidemiology and prevention. Vasc Health Risk Manag.

[14] Boot E, Ekker MS, Putaala J, Kittner S, De Leeuw FE, Tuladhar AM (2020). Ischaemic stroke in young adults: a global perspective. J Neurol Neurosurg Psychiatry.

[15] Yahya T, Jilani MH, Khan SU (2020). Stroke in young adults: current trends, opportunities for prevention and pathways forward. Am J Prev Cardiol.

[16] El-Hajj M, Salameh P, Rachidi S, Hosseini H (2016). The epidemiology of stroke in the Middle East. Eur Stroke J.

[17] Al-Jishi AA, Mohan PK (2000). Profile of stroke in Bahrain. Neurosci J.

